# The spatial revolution in immuno-oncology: artificial intelligence decoding NK cell niches to predict therapeutic response

**DOI:** 10.3389/fimmu.2026.1915941

**Published:** 2026-08-03

**Authors:** Yun-qiu Gao, Tong Liu, Yang Yang

**Affiliations:** 1 Department of Dermatology, The First Hospital of China Medical University, Shenyang, Liaoning, China; 2 Key Laboratory of Immunodermatology, Ministry of Education and National Health Commission of the People's Republic of China (NHC), Shenyang, Liaoning, China; 3 National Joint Engineering Research Center for Theranostic of Immunological Skin Disease, Shenyang, Liaoning, China

**Keywords:** artificial intelligence, biomarkers, natural killer cells, spatial transcriptomics, tumor microenvironment

## Abstract

Natural killer (NK) cells are important effector cells of the innate immune system and have been investigated as a therapeutic platform for cancer immunotherapy. Although NK cell-based therapies have shown clinical activity in hematological malignancies, their application in solid tumors remains limited by restricted tumor infiltration, functional suppression, and the complexity of the tumor microenvironment (TME). Recent advances in spatial transcriptomics and artificial intelligence (AI) have provided new approaches for characterizing NK cell distribution, functional states, and cellular interactions within the TME. Critically, the spatial distribution and structural organization of NK cells within tumor niches — including their proximity to tumor cells, stromal barriers, and immune effector partners — are fundamental determinants of their cytotoxic function. A deeper understanding of how spatial context shapes therapeutic response is therefore central to advancing NK cell immunotherapy. This review summarizes how AI-assisted analysis of spatial and multi-omics data may contribute to the discovery and validation of biomarkers associated with NK cell therapy response. It also discusses the potential applications of AI in optimizing chimeric antigen receptor (CAR)-NK cell engineering, combination therapy strategies, and individualized dosing regimens. By synthesizing studies published in recent years, this review highlights the emerging shift from response prediction toward treatment optimization, while emphasizing the current limitations of available evidence, including model interpretability, data heterogeneity, causal inference, and clinical validation. Finally, we discuss how four-dimensional (4D) dynamic monitoring and explainable AI may support the future development of more precise and personalized NK cell immunotherapy strategies.

## 1 Introduction

Natural killer (NK) cells, as important effector cells of the innate immune system, have been investigated as a therapeutic platform for cancer immunotherapy after T cells, owing to their capacity to recognize and eliminate tumor cells without prior sensitization (1, 2). In recent years, therapeutic strategies exemplified by chimeric antigen receptor (CAR)-modified NK cells and adoptive NK cell infusion have shown encouraging clinical outcomes in hematological malignancies. For instance, in relapsed/refractory non-Hodgkin lymphoma, CD19 CAR-NK cell therapy achieved an objective response rate (ORR) of 78%, with a complete response (CR) rate of 56% (3, 4). Compared with CAR-T cell therapy, NK cell therapy may offer certain safety and accessibility advantages, including a lower reported risk of cytokine release syndrome (CRS) and neurotoxicity, as well as the potential for “off-the-shelf” application without strict HLA matching (5, 6).

Nevertheless, NK cell therapy, particularly in the context of solid tumors, still faces several challenges. First, adoptively infused NK cells often exhibit limited *in vivo* persistence and proliferative capacity, which may restrict durable anti-tumor effects (7, 8). Second, the immunosuppressive tumor microenvironment (TME) of solid tumors can limit NK cell infiltration and functional activity through physical barriers, inhibitory cell populations such as regulatory T cells and tumor-associated macrophages, and metabolic stresses including hypoxia and acidic conditions (9, 10). Mechanistically, metabolic stresses such as hypoxia (via HIF-1α) and acidic pH directly impair NK cell recognition and cytotoxicity. Furthermore, immunosuppressive cells like Tregs and M2-TAMs secrete inhibitory factors (e.g., TGF-β, IL-10) and create physical barriers restricting NK cell trafficking. These factors contribute to the limited efficacy of NK cell therapy in solid tumors compared with hematological malignancies. A further challenge in current clinical practice is how to accurately predict and stratify patients who are more likely to benefit from NK cell therapy. Conventional biomarkers, such as PD-L1 expression and tumor mutational burden, have limited predictive capacity for NK cell therapeutic efficacy (11, 12). Importantly, these conventional markers primarily reflect T cell-mediated adaptive immunity and are fundamentally insufficient for NK cell-based therapies: PD-L1 expression captures the PD-1/PD-L1 axis relevant to T cell exhaustion, whereas NK cell cytotoxicity is governed by a distinct set of activating and inhibitory receptors; similarly, TMB reflects the neoantigen load relevant to T cell recognition, but NK cells recognize stress-induced ligands and MHC class I loss rather than neoantigens. Therefore, NK-specific biomarkers are urgently needed, including the dynamic balance of activating receptor-ligand interactions (e.g., NKG2D–MICA/B, NKp30–B7-H6) and inhibitory receptor-ligand interactions (e.g., KIR–HLA-C, NKG2A–HLA-E), as well as the spatial distribution of NK cell subsets within the TME, which collectively provide a more biologically relevant framework for predicting NK cell therapeutic efficacy. This limitation may lead to inefficient allocation of therapeutic resources and delayed treatment opportunities for potential responders. Therefore, developing biomarkers capable of predicting treatment outcomes and supporting patient stratification remains an important issue in the further development of NK cell therapy.

**Figure 1:**
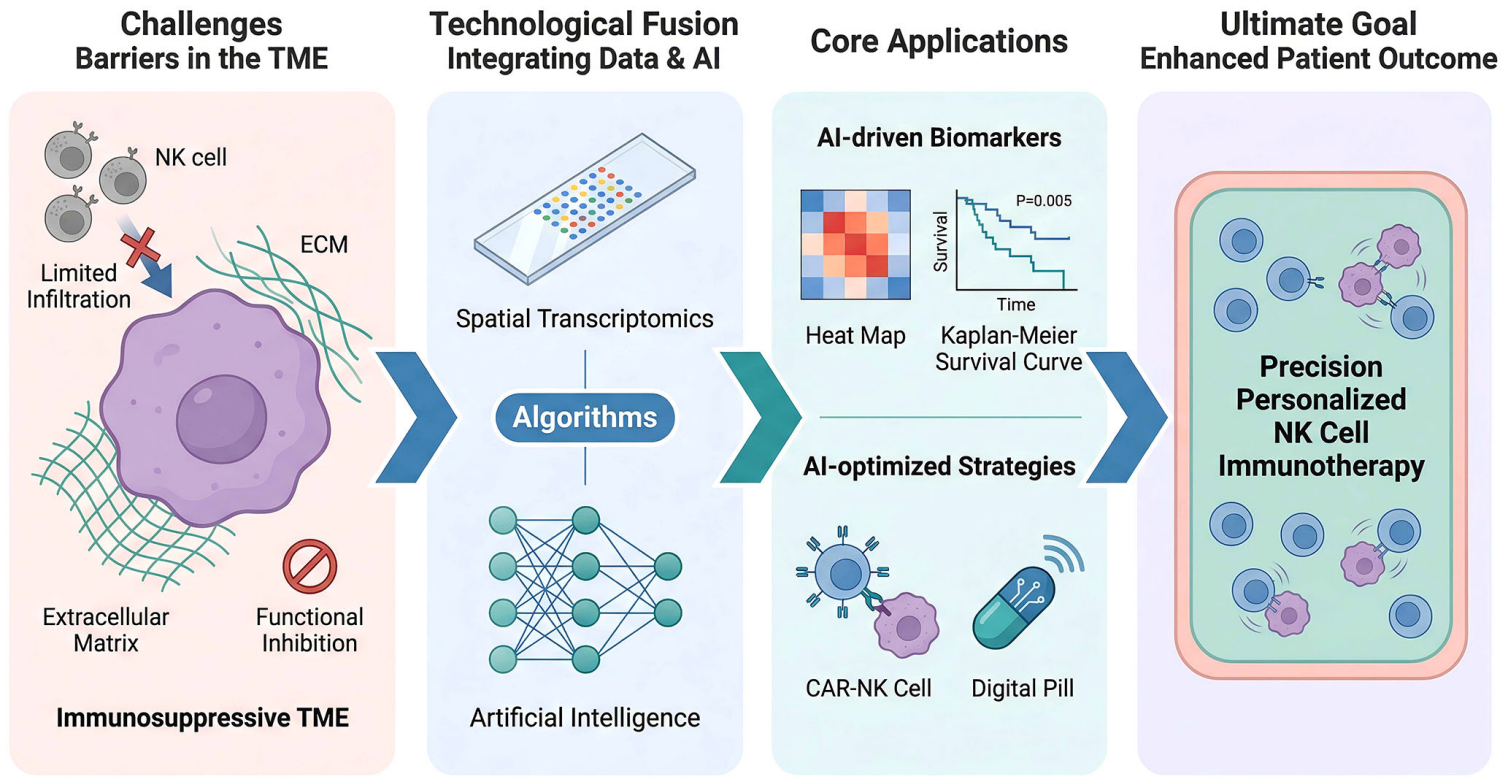
Spatial revolution in immuno-oncology: AI decoding NK cell niches. The illustration outlines the transition from high-dimensional spatial multi-omics data integration to decoding TME architecture. It highlights the dual function of AI: discovering novel spatial biomarkers for prognosis and guiding systemic therapeutic strategies (including CAR-NK design, combination therapies, and dynamic dosing regimens).

Artificial intelligence (AI) technologies provide potential approaches to addressing some of these challenges. By integrating high-dimensional clinical data, multi-omics data, including genomics, transcriptomics, and proteomics, and histopathological image data, AI models may help identify complex patterns that are difficult to capture using conventional statistical methods, thereby supporting biomarker discovery and treatment strategy optimization (13, 14). For example, machine learning-based models have been reported to predict NK cell cytotoxic efficacy with an accuracy of 84.2% and an area under the curve (AUC) of 0.908 (15). In this context, this review summarizes how AI may contribute to the development of next-generation NK cell immunotherapy. As outlined in the framework of **Figure 1**, this review follows the logical sequence of TME-related challenges, the technical integration of spatial multi-omics and AI, and potential clinical applications. It focuses on two main aspects: first, how AI-assisted analysis of high-dimensional data, including spatial transcriptomics, may facilitate the discovery of spatial biomarkers associated with therapeutic response; and second, how AI may support the optimization of treatment strategies, including CAR-NK structural design, donor selection for adoptive infusion, and combination therapy formulation, thereby contributing to more precise and personalized NK cell immunotherapy strategies (16, 17).

## 2 AI-driven biomarker discovery for NK cell therapy: decoding the spatial niche

Accurately predicting which patients stand to benefit from NK cell therapy is central to maximizing its clinical value and preventing resource misallocation. Conventional biomarkers demonstrate limited predictive capacity in this context, whereas AI opens new avenues for discovering novel and high-performance biomarkers by integrating high-dimensional multi-omics data (13, 14, 18). These biomarkers include non-spatial features derived from peripheral blood or tissue samples. More importantly, the integration of AI with spatial technologies enables the decoding of the “architectural” characteristics of the TME. This approach may reveal spatially informed biomarkers (19, 20).

### 2.1 Spatial biomarkers

Spatial omics technologies provide a panoramic view for dissecting immune cell behavior *in situ* within tissues, while AI may facilitate the interpretation of these complex spatial landscapes. Their integration not only provides a potential explanation for the “NK cell paradox,” but also illuminates a fundamental principle in tumor immunity: “where you are” frequently matters more than “what you are” (21, 22). In this context, spatial biomarkers should not be understood merely as anatomical annotations of immune infiltration, but as multidimensional indicators that integrate cell identity, localization, functional state, and intercellular communication. Crucially, the spatial distribution and organization of NK cells within the TME — including their localization relative to the tumor invasive margin, their proximity to antigen-presenting cells, and their distance from immunosuppressive populations — fundamentally shape their functional state and determine the overall therapeutic response. This spatial context is therefore not merely an anatomical annotation but a biologically active determinant of NK cell efficacy.

A representative example comes from hepatocellular carcinoma (HCC), in which NK cell infiltration often shows a weak or inconsistent correlation with patient prognosis. This phenomenon is difficult to explain using conventional immunological evaluation frameworks that primarily quantify immune cell abundance. Jia et al. (2025) employed spatial transcriptomics to demonstrate that the critical determinant of HCC recurrence is not the absolute number of NK cells, but their spatial distribution pattern and functional state (23). Specifically, the SPON2+ NK cell subset localized at the tumor invasive margin (IF) exhibits pronounced anti-tumor activity, whereas NK cells infiltrating the tumor core (TC) show a loss of effector function. Based on this spatial logic, the TIMES scoring system was constructed to integrate NK cell localization and immune microenvironmental features. In a validation cohort of 231 patients, it achieved a predictive accuracy of 82.2%. Regarding prognostic performance, the TIMES system yielded an area under the curve (AUC) of 0.82, significantly outperforming conventional TNM staging (AUC = 0.51) and BCLC staging (AUC = 0.53) (23). These findings suggest that the inconsistent clinical interpretation of NK cell infiltration may partly reflect spatial heterogeneity rather than biological irrelevance of NK cells themselves.Nevertheless, the generalizability of the TIMES scoring system to other “cold” tumors such as pancreatic cancer and prostate cancer warrants further validation. This study also underscores the importance of spatial localization.

This evidence shifts the analytical focus from simple infiltration density to the mechanisms that determine spatial recruitment and functional positioning. However, translating this insight into therapeutic intervention remains challenging. Although strategies such as chemokine engineering and CAR-NK cell design may help redirect NK cells toward tumor-relevant regions, their feasibility and durability in heterogeneous tumor microenvironments require further evaluation (19, 24).

Moreover, whether the TIMES scoring system can be generalized to other immunologically “cold” tumors, such as pancreatic cancer and prostate cancer, remains to be validated. Therefore, the major significance of this study is not only the identification of a specific HCC-associated spatial biomarker, but also the establishment of a broader conceptual framework in which NK cell function is interpreted through spatial context.

**Figure 2:**
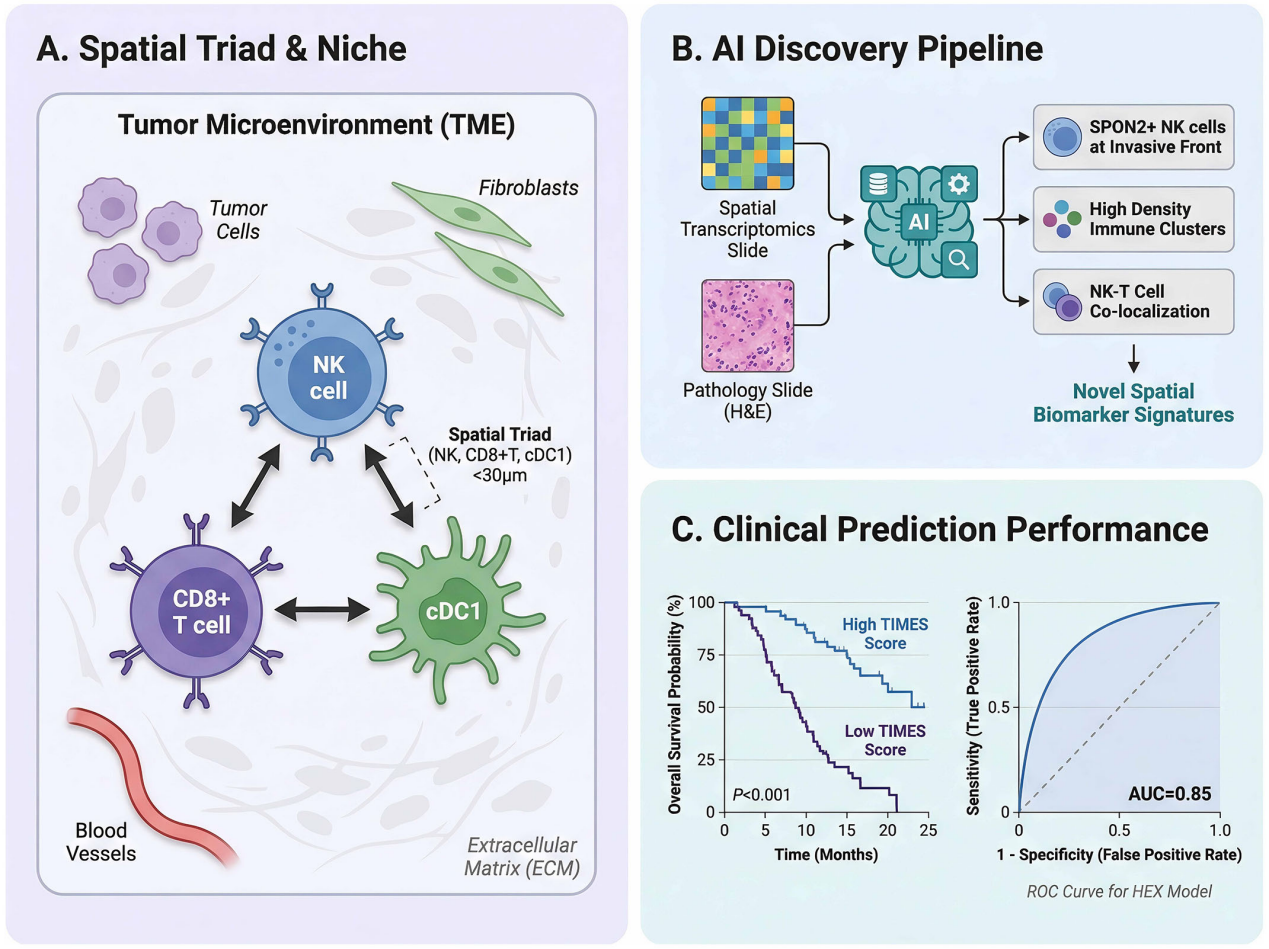
Al-driven spatial biomarkers in NK cell therapy. **(A)** Spatial topological model illustrating the immune-activating “triad” structure (NK cells, CD8+ T cells, and cDC1 cells) within a critical proximity (< 30 μm). **(B)** Workflow of the TIMES scoring system utilizing spatial transcriptomics to stratify risk based on SPON2+ NK cell localization at the invasive margin. **(C)** HEX model evaluating the predictive performance of virtual spatial omics generated directly from H&E-stained slides, showing superior response prediction (AUC = 0.85) compared to traditional PD-L1 testing.

Beyond the localization of NK cells themselves, effective anti-tumor immune responses depend on the precise spatial coordination of multiple immune cell populations. Wessel et al. (2024) revealed that in non-small cell lung cancer (NSCLC), the spatial proximity of NK cells and CD8+ T cells (< 30 μm) serves as a key predictor of patient survival benefit (HR = 0.65) (25). This spatial cluster is composed of NK cells, CD8+ T cells, and cDC1 antigen-presenting cells, forming a core “triad” structure associated with immune activation (26). This spatial topological model is depicted in **Figure 2A**. Ehlers et al. observed an analogous cooperative pattern in HER2+ breast cancer (27). These findings indicate that the clinical efficacy of NK cells depends not only on their infiltration level or functional state, but also on the efficiency of their spatial interactions with other effector populations. Nevertheless, current spatial analyses largely rely on static cross-sectional data, limiting their capacity to capture the dynamic evolution of spatial relationships during treatment. For example, it remains unclear how immune checkpoint inhibitors reshape the spatial proximity between NK cells and CD8+ T cells, and whether such spatial remodeling is causally linked to treatment response. Longitudinal spatial tracking and causal inference modeling may help address these questions. In addition, the biological universality of the “optimal distance” threshold, such as 30 μm, remains uncertain across distinct tumor microenvironmental contexts, and it is still unclear whether this statistical cutoff corresponds to a functional immune-interaction threshold.

Although spatial omics provides high-resolution biological information, its high cost and operational complexity remain major bottlenecks for large-scale clinical adoption. AI-based histopathological inference — specifically, deep learning models applied to routine histopathology images — may offer a practical route to overcome this limitation. These approaches include convolutional neural networks (CNNs) and vision transformers for feature extraction from H&E slides, graph-based modeling of cellular spatial networks to quantify immune cell interactions, and multimodal integration frameworks that combine imaging data with transcriptomic or proteomic profiles. Each of these approaches addresses specific biological questions: deep learning for histopathology enables the inference of molecular spatial features without costly assays; graph-based models can quantify the topology of immune cell neighborhoods relevant to NK cell function; and multimodal integration links spatial patterns to clinical outcomes such as immunotherapy response. The HEX deep learning model developed by Li et al. (2026) reconstructs the spatial distribution of 40 immune proteins, including the NK cell marker NKp46, directly from routine hematoxylin and eosin (H&E)-stained sections, achieving a correlation coefficient of r > 0.75 (28). In a cohort of 2,298 NSCLC patients, the immune checkpoint inhibitor (ICI) response prediction model based on virtual spatial features achieved an AUC of 0.85, significantly outperforming clinically established PD-L1 detection (AUC ≈ 0.65) (28). This advance demonstrates the potential of deep learning to extract latent molecular and spatial information from histomorphological images, thereby lowering the technical threshold for spatial analysis. Nevertheless, virtual spatial omics faces several challenges. First, the “black-box” nature of deep learning models may reduce biological interpretability and clinical trust. Second, model generalizability is constrained by the diversity of training data, including potential racial and population differences, and its robustness across heterogeneous populations therefore requires further validation (29, 30). Third, the field currently remains focused mainly on diagnostic and prognostic prediction. Further work is needed to determine whether spatial pattern recognition can guide clinical intervention strategies (31).

### 2.2 Non-spatial biomarkers

In contrast to spatial biomarkers, which focus on decoding the architectural organization of tissues, non-spatial biomarkers prioritize the assessment of the intrinsic functional state of the tumor immune microenvironment and its dynamic evolution over time. In this domain, AI can support the integration of multidimensional omics data. It may also help extract weak predictive signals from high-background noise. Another potential application is the development of continuous dynamic monitoring, rather than relying only on static single-timepoint assessments.

NK cell-mediated cytotoxicity is fundamentally governed by the precise balance between activating and inhibitory receptor signaling. Conventional single-molecule biomarkers, such as NKG2D ligands, fail to comprehensively capture the complex regulatory networks arising from multi-receptor cooperation, and this single-indicator evaluation framework limits the accurate assessment of NK cell killing efficacy. This limitation is compounded by the fact that conventional biomarkers such as PD-L1 and TMB, which were designed to predict T cell-based immunotherapy responses, fail to capture the complex receptor-ligand biology governing NK cell activation. NK cells integrate signals from multiple activating receptors (NKG2D, NKp30, NKp44, NKp46, DNAM-1) and inhibitory receptors (KIR family, NKG2A, TIGIT, PD-1) simultaneously, and the net cytotoxic output is determined by the dynamic balance of these competing signals. Consequently, NK-specific biomarkers that capture this receptor-ligand interplay are more biologically appropriate for predicting NK cell therapy outcomes. The random forest model developed by Ma et al. integrates the expression profiles of 11 receptor–ligand pairs, elevating the predictive accuracy of NK cell treatment response to 84.2% (AUC = 0.908) (15). The core contribution of this model lies in quantifying the nonlinear interactions between receptors and ligands. The study reveals that the prognostic value of high expression of specific activating ligands may be nullified by the concurrent activation of inhibitory receptors — a complex compensatory mechanism that only machine learning algorithms can precisely resolve. However, this model has primarily been validated in ovarian cancer cohorts, and its generalizability to hematological malignancies requires further evaluation. For example, the 8-gene prognostic model constructed by Zhao et al. in multiple myeloma (32) demonstrates preliminary potential, yet the substantial heterogeneity between its gene signature and solid tumor models suggests that tumor type specificity remains a critical bottleneck constraining the generalizability of multi-omics predictive models. Future research should focus on constructing universal predictive frameworks applicable across cancer types, or on leveraging transfer learning to enable rapid model adaptation across different tumor types.

Peripheral blood biomarkers offer the advantages of non-invasiveness and repeatability, providing a feasible approach for tracking the longitudinal evolution of immune status. Choi et al. (2020) demonstrated that baseline peripheral blood NK cell activity (≥ 1,200 pg/mL) predicts immunotherapy response in NSCLC with a sensitivity of 80% (11), confirming the clinical value of peripheral blood NK cells as an independent prognostic indicator. Of greater clinical significance is the concept of “circulating immune cell dynamics” proposed by Rubio et al. (2023) (33): treatment responders exhibit a significant increase in peripheral blood NK cell frequency at four weeks post-administration, whereas non-responders display a declining trend. This dynamic trajectory reveals the predictive role of early-phase immune remodeling for long-term therapeutic outcomes. Furthermore, the ratio of NK cells to granulocytic myeloid-derived suppressor cells (NMR) outperforms conventional PD-L1 expression in predictive performance, suggesting that the benefit of immunotherapy depends not solely on the baseline abundance of effector cells such as NK cells, but critically on the antagonistic balance between effector and suppressive cell populations. Nevertheless, current studies predominantly focus on correlational analyses, and the underlying causal mechanisms driving NK cell frequency fluctuations remain unclear. Future research should incorporate refined time-series analysis and causal inference algorithms to elucidate the mechanistic link between peripheral blood immune dynamics and TME remodeling.

Soluble inhibitory factors within the TME constitute a critical mechanistic barrier limiting NK cell efficacy. Švubová et al. found that TGF-β not only directly suppresses NK cell cytotoxicity, but also impairs NK cell infiltration into tumors by downregulating chemokine receptor expression (34). This indicates that pre-treatment peripheral blood TGF-β levels may serve as an important negative predictive factor for prognosis. However, cytokine networks exhibit a high degree of complexity and compensatory redundancy. For instance, the inhibitory effects of TGF-β may be partially counteracted by activating signals from IL-5 or IL-2. The net effect of such multi-signal integration can only be accurately captured through systematic multi-cytokine profiling. In addition, the correspondence between peripheral blood cytokine levels and the local TME state requires systematic validation through paired tissue–blood sample studies. The value of AI in this domain extends beyond identifying high-risk patients to supporting clinical decision-making—for example, identifying the subpopulations most likely to benefit from combination therapies such as TGF-β inhibitors.

Surveying the current landscape of non-spatial biomarker research, the primary bottleneck lies in the fact that model functionality is largely confined to efficacy prediction, lacking the capacity to guide therapeutic intervention (35) Although existing models effectively identify high-risk patients with low NK cell activity, they have yet to provide optimized pathways for enhancing immune responses in these populations. Achieving the transition from static prediction to dynamic intervention represents the central task of future intelligent immunotherapy. AI models should evolve from simply answering “who will respond” to answering “how to induce a response.” This requires the deep integration of technologies such as reinforcement learning and causal inference into biomarker research (36, 37), constructing active decision-support systems capable of assisting clinicians in formulating personalized dosing regimens, combination therapy strategies, and cellular engineering designs (a comprehensive comparison between conventional methods and AI-driven biomarkers is summarized in **Table 1**).

**Table 1:** Multidimensional comparison between traditional and AI-driven spatial biomarkers in NK cell immunotherapy.

Comparison dimension	Traditional biomarker paradigm	AI-driven spatial/dynamic paradigm	Representative studies & core findings	Clinical translation bottlenecks
Evaluation Core	Static, single-molecule expression or absolute cell density	Spatial topology, cellular neighborhood interactions, and longitudinal dynamics	TIMES score quantifies SPON2+ NK cell spatial distribution to predict HCC recurrence (23). Dynamic NK cell trajectories predict NSCLC immunotherapy response (33).	Lack of standardized spatial metrics and biological cut-off values.
Information Resolution	Linear correlation assumption (High infiltration = Expected high response)	Non-linear complex networks (Receptor-ligand compensation, multi-cell synergy)	ML model based on receptor-ligand interactions predicts NK cytotoxicity (15). Spatial “triad” (NK, CD8+ T, cDC1) predicts survival benefits (25).	“Black box” nature of deep learning models limits biological interpretability.
TME Characterization	Discrete “parts list” (Ignores physical barriers and spatial heterogeneity)	Structured “functional ecosystem” (Identifies immune-activating/suppressive niches)	HEX virtual proteomics predicts 40 immune proteins’ spatial distribution directly from H&E slides (28).	2D slice sampling bias; high costs of spatial multi-omics technologies.
Clinical Utility	Passive prediction (Binary classification of responders vs. non-responders)	Active intervention guidance (Predicts response and suggests spatial remodeling targets)	Reinforcement learning simulates TME dynamics to optimize combination treatment schedules (38).	Requires large-scale prospective validation; immature regulatory pathways for AI software.

Paradigm shift from traditional static evaluation to AI-driven spatial and dynamic approaches in assessing TME and guiding clinical decisions.

## 3 AI-optimized NK cell therapeutic strategies

If biomarkers serve as the diagnostic tools for assessing tumor immune status, then the optimization of therapeutic strategies represents the means of intervening in that status. AI may improve the efficiency of existing NK cell therapeutic regimens. It may also help address some limitations of conventional trial-and-error approaches. In this context, AI-based methods — including multimodal integration of genomics and spatial transcriptomics data, deep learning for histopathological pattern recognition, graph neural networks for modeling cellular interaction networks, and reinforcement learning for adaptive treatment scheduling — support a more data-driven approach to treatment design and optimization. These methods address specific clinical questions: multimodal integration helps identify which patients harbor NK-permissive TME architectures; graph-based models reveal the spatial interaction networks that predict NK cell functional states; and reinforcement learning enables dynamic dose adjustment based on real-time immune monitoring. This section examines how AI reshapes the future of NK cell immunotherapy across three dimensions: cellular engineering, combination therapy strategies, and dosing regimen design.

### 3.1 AI-driven NK cell engineering

To clarify the translational readiness of these approaches, we categorize them into (a) validated applications, (b) early proof-of-concept, and (c) purely conceptual proposals.

**Figure 3:**
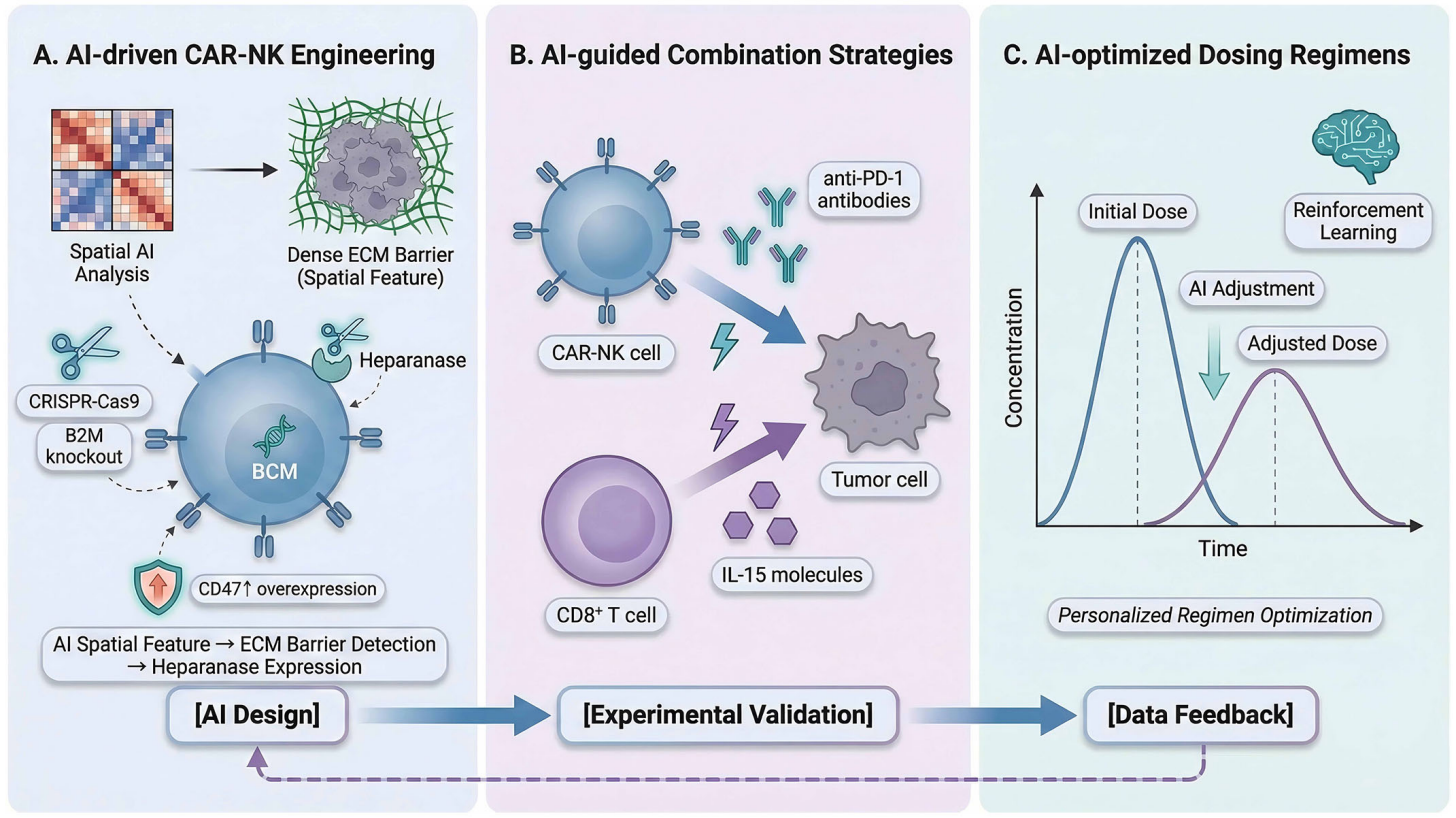
AI design - experimental validation - data feedback. **(A)** Multiplex gene editing strategies for CAR-NK cells utilizing patient-specific profiles to optimize targets such as B2M, HLA-E, and CD47. **(B)** AI-driven synergistic combination therapy, integrating NK-specific checkpoints with T cell inhibitors. **(C)** Reinforcement learning (RL) framework for dynamic, pulsatile dosing schedules using multiscale mathematical modeling.

(a) Validated applications: The design of CAR-NK cells is a high-dimensional optimization problem. Antigen selection, co-stimulatory domains, gene-editing targets, and vector platforms each involve multiple options. The interactions among these choices are often nonlinear (39–41). The conventional one-variable-at-a-time trial-and-error strategy is not only inefficient, but also risks missing the optimal combination. Shifting from immune evasion to precision stealth, allogeneic CAR-NK cells face the central challenge of host immune rejection (42). Kim et al. systematically delineated a multi-gene editing strategy: B2M knockout eliminates HLA class I expression to evade T cell attack, HLA-E overexpression suppresses recognition by host NK cells, and CD47 overexpression blocks macrophage phagocytosis (43). However, excessive immune evasion may compromise the intrinsic function of the engineered NK cells. AI may help integrate patient-specific HLA/KIR background data to predict suitable gene-editing combinations. This concept is illustrated by the AI-driven multi-gene editing and spatial feature-responsive design in **Figure 3A**. Such approaches could support the development of more customized CAR-NK cell products.

The clinical trial published by Marin et al. in Nature Medicine (2024) provides support for this strategy. In this study, 37 patients with CD19+ B cell malignancies received CAR19/IL-15 NK cell therapy. The overall response rate was 48.6% at both day 30 and day 100, and the one-year overall survival rate was 68%. No CRS, neurotoxicity, or graft-versus-host disease (GVHD) was observed (44) [40].The study highlights the influence of donor selection on therapeutic outcomes. CAR-NK cells derived from optimal cord blood units showed superior functionality and persistence. These optimal units were characterized by nucleated red blood cells ≤ 8 × 10^7^ and a collection-to-cryopreservation interval ≤ 24 hours. These findings suggest that AI may be useful for donor selection and quality prediction. Conventional strategies that enhance NK cell function by overexpressing activating receptors or knocking out inhibitory receptors may not fully account for the heterogeneity of the TME (45). Recent studies have therefore explored more context-dependent approaches to modulating NK cell activation. Bexte et al. demonstrated that CRISPR/Cas9-mediated knockout of NKG2A significantly enhances the cytotoxicity of CAR-NK cells against multiple myeloma (46). A genome-wide CRISPR screen published by Biederstädt et al. in Cancer Cell (2025) identified key regulators of NK cell function, providing a target resource for cellular engineering (45). The advantage of AI lies in integrating tumor multi-omics data to predict the optimal enhancement strategy for specific tumor types, and even to design intelligent activation systems that respond to TME signals.

(b) Early proof-of-concept: Regarding vector platforms, non-viral engineering strategies are gaining momentum. Bexte et al. (2024) successfully engineered CAR-NK cells using the Sleeping Beauty transposon system, circumventing the insertional mutagenesis risk associated with viral vectors while demonstrating efficient gene integration safety and anti-tumor efficacy (41). Shankar et al. employed virus-free CRISPR knock-in technology to simultaneously disrupt inhibitory signaling and express CAR in primary NK cells with high precision (47). (c) Purely conceptual proposals: Nevertheless, a shared limitation of current AI applications is that they primarily seek optimal solutions within existing design spaces rather than exploring entirely new ones (40). Whether AI can design immune evasion mechanisms that transcend the existing HLA system remains an open question — one that necessitates a shift from supervised learning to generative AI. Furthermore, most training data currently originate from *in vitro* experiments and may not fully recapitulate *in vivo* complexity. The off-target effects and long-term safety of multi-gene editing also remain uncertain. In addition, patient-specific HLA/KIR polymorphism may increase the cost and time required for implementing a personalized “one patient, one regimen” approach (42). Future breakthroughs will require the deep integration of AI with high-throughput screening and organoid models, establishing a closed-loop iterative cycle from AI-guided design to experimental validation and data feedback.

### 3.2 AI-guided combination therapy strategies

Similar to cellular engineering, AI-guided combination strategies span a spectrum of translational readiness, from validated applications to purely conceptual proposals.

(a) Validated applications and (b) Early proof-of-concept: The therapeutic efficacy of NK cell therapy is frequently constrained by the TME — immune checkpoints, immunosuppressive cells, and metabolic barriers collectively constitute an immune evasion network (48, 49). Recent advances in AI-guided cancer immunotherapy and nanotechnology-driven precision medicine offer complementary strategies to address these barriers. AI-enhanced synergistic chemo-immunotherapy has demonstrated that chemotherapy-induced immunogenic cell death can remodel “cold” tumors into “hot” tumors, potentiating immune checkpoint inhibitor efficacy, and that AI-driven multi-omics integration can identify optimal chemo-immunotherapy combinations and predict treatment responses with greater precision (50). Furthermore, AI-driven liposomal nanocarriers represent an emerging platform for enhancing cancer immunotherapy by enabling targeted drug delivery, controlled release, and improved modulation of the TME, thereby overcoming immunosuppressive barriers that restrict NK cell function (51). Single NK cell infusion struggles to overcome this network, yet the number of possible combination regimens grows exponentially. How can the optimal strategy be identified from this combinatorial explosion? AI offers a new perspective, as illustrated by the AI-guided antibody and cytokine synergy strategy in **Figure 3B**. Conventional combination therapy design often relies on simplified biological assumptions and may overlook complex interactions among tumor cells, immune cells, and therapeutic agents. AI-based methods may provide a more data-driven approach for identifying candidate combinations. Strassheimer et al. combined local CAR-NK cell injection with anti-PD-1 therapy in glioblastoma. This combination enhanced anti-tumor effects and induced NKT cell responses. These findings suggest a potential strategy for treating “immune-cold” tumors[49]. Khan et al. systematically elaborated on the “dual unlocking” value of combining NK cell-specific checkpoints (e.g., KIR and NKG2A) with T cell checkpoint inhibitors (48). Lin et al. elucidated the fine-tuned regulatory mechanisms of inhibitory receptors such as NKG2A/CD94 on the NK cell surface and their interactions with the tumor ligand HLA-E (49). AI-based analysis may help identify candidate synergistic combinations. It may also be useful for screening potentially antagonistic interactions, although such predictions require experimental validation. For example, a clinical trial of ANKTIVA (an IL-15 superagonist) combined with ICI therapy in NSCLC demonstrated a 12-month overall survival rate of 57% and an 18-month overall survival rate of 34%, significantly outperforming ICI monotherapy.

Nevertheless, current AI models confront challenges related to data quality and data bias. Most combination therapy data originate from retrospective studies that lack standardized assessments and detailed immune monitoring. A “positive result bias” in published data may cause AI models to overestimate success rates (52). Because the TME evolves dynamically during treatment, fixed combination regimens may not adequately capture changes in immune suppression, tumor adaptation, and therapeutic sensitivity. This supports the need to explore more adaptive combination strategies. (c) Purely conceptual proposals: Reinforcement learning offers the possibility of adaptive combination therapy — by monitoring immune status in real time and dynamically adjusting the combination regimen. However, real-time monitoring is costly and lacks standardized platforms; reinforcement learning requires extensive trial-and-error data, and clinical “trial and error” implies that patients may receive suboptimal treatment. Realizing this vision requires the deep integration of clinical trial design with AI algorithms, adopting a “Bayesian adaptive design” to strike a balance between exploration and exploitation. A key challenge is bridging the gap between *in vitro* and *in vivo* synergy. Current AI models often predict combination therapy outcomes based on *in vitro* data. However, *in vitro* synergy does not necessarily translate into *in vivo* synergy. *In vitro* experiments fail to recapitulate the complexity of the *in vivo* TME — factors such as the spatial distribution of immune cells, metabolic gradients, and the physical barriers of the extracellular matrix are all neglected. More strikingly, Galvez-Cancino et al. found that NK cell abundance may influence ICI efficacy, and that in certain contexts NK cells may even impede CD8+ T cell-mediated immunity (53). Ma et al. further explored the complex dual role of NK cells as natural killers versus natural regulators (54). These findings caution that combination therapy design cannot simply assume that “more immune cells equals better efficacy.” Furthermore, the additive toxicity of combination therapies is frequently underestimated. Kohanbash et al. found that immune checkpoint blockade can trigger NK cell chemokine release and recruit CD8+ T cells to enhance immunity. However, this process may also give rise to over-activation-related toxicity (55). Future AI models will need to integrate pharmacokinetic, pharmacodynamic, and systems biology models to achieve joint optimization of efficacy and toxicity.

### 3.3 AI-optimized dosing regimens and dose design

Dosing optimization can similarly be stratified by its proximity to clinical implementation.

(a) Validated applications and (b) Early proof-of-concept: Dose design for cell therapies is more complex than dose design for small-molecule drugs. In this context, dose refers not only to cell quantity. It also includes cell quality, timing, and infusion frequency (56). The conventional “3 + 3” dose-escalation design fails to account for patient heterogeneity. Rather than focusing only on infused cell quantity, the random forest algorithm proposed by Wang et al. examined exposure–response relationships. The model integrated multidimensional data on cell expansion, persistence, toxicity, and efficacy (57). AI can identify ineffective and excessively toxic doses early in the trial, allocating more patients to “potentially efficacious doses.” This “seamless Phase I/II design” improves trial efficiency and aligns with the requirements of the FDA Project OPTIMUS. However, the core assumption that cell expansion positively correlates with efficacy does not always hold — excessive expansion may lead to cellular exhaustion. Furthermore, the definition of dose itself remains contentious: does the cell count at the time of infusion matter more, or the peak cell count following *in vivo* expansion?

Compared with fixed dosing strategies, the multi-scale mathematical model combined with a reinforcement learning framework developed by Liu et al. explored the possibility of dynamic dose adjustment. The model simulated dynamic interactions between tumors and the microenvironment to optimize combination therapy schedules (38). This reinforcement learning-based pulsed adjustment process is detailed in **Figure 3C**. This study employs a parallel actor-critic algorithm to predict optimal regimens, validating the dynamic combination strategy in a glioblastoma model. Banumathi et al. systematically reviewed the application of reinforcement learning in personalized medicine, noting its capacity to achieve an optimal balance between tumor control and toxicity (37). Nevertheless, real-time monitoring remains costly and lacks standardization; the black-box nature of reinforcement learning may limit acceptance among clinicians. Regarding AI-driven innovation in clinical trial design, Rosenthal et al. proposed a dynamic deployment framework, emphasizing that trial design must accommodate the continuous updating of AI models (58). Azenkot et al. highlighted the promise of AI-driven approaches in enhancing clinical trial design and supporting adaptive methods for precision medicine (59). The CURATE.AI platform developed by Blasiak et al. leverages each patient’s own small dataset to dynamically personalize dose recommendations, achieving genuine individualized optimization (60). The highly cited review by Johnson et al. underscores the importance of integrating clinical, genomic, and phenotypic data for treatment planning (56).

(c) Purely conceptual proposals: Currently, AI is primarily applied to optimize existing dosing paradigms (e.g., dose escalation and fixed-interval infusion) rather than to explore entirely new ones. Can AI design pulsed dosing regimens — concentrating NK cell infusions during periods of minimal tumor immunosuppression? Or stratified dosing — first administering a low-dose “reconnaissance” infusion to assess the TME, then adjusting subsequent doses accordingly? These novel paradigms may require the deep integration of generative AI and reinforcement learning. Furthermore, current AI models are trained on population-level data, whereas truly individualized dose design requires the integration of each patient’s genomic, immunomic, and metabolomic data. Achieving individualized predictions under conditions of limited data may necessitate transfer learning and few-shot learning techniques.

### 3.4 AI-Driven causal inference for optimizing precision NK cell therapy

Current deep learning models have shown strong performance in predicting NK cell therapy responses. However, most of these models rely on correlational analyses. They often struggle to elucidate the complex biological causal chains within the TME, which may limit the transparency of clinical decision-making. As AI-driven medicine enters the causal era, leveraging causal inference techniques to identify the core factors governing NK cell function has become essential for achieving precision intervention. By integrating structural causal models (SCMs) with counterfactual reasoning, researchers can identify critical causal nodes from multi-omics data. For example, neural network-based causal discovery algorithms such as DAG-GNN may be used at specific spatial loci. These algorithms can help infer whether cytokine secretion or physical barriers play a more direct causal role in restricting NK cell infiltration (25, 28). Furthermore, causal inference empowers AI to conduct virtual intervention experiments at the preclinical stage: by simulating counterfactual outcomes under different intervention conditions, it precisely predicts changes in NK cell killing efficiency following the blockade of specific immune checkpoints, thereby guiding the rational selection of combination therapies and preventing ineffective interventions caused by shared confounding factors. More importantly, causal representation learning may enhance the causal robustness of AI models. This approach could help NK cell response models trained on specific cohorts remain informative when applied to clinically heterogeneous samples. It may also help address the out-of-distribution (OOD) generalization challenge that deep learning models face in cross-center validation. A summary of the core applications and limitations of AI across the NK cell therapy optimization strategies discussed above is provided in **Table 2** (23).

**Table 2:** AI-empowered optimization for next-generation NK cell therapy strategies.

Optimization dimension	Traditional experience-driven limitations	AI-driven solutions & algorithms	Recent breakthroughs	Future evolutionary pathways
CAR-NK Engineering	Inefficient trial-and-error; immune evasion pursuit impairs intrinsic NK function.	Multi-omics predictive models; data mining of CRISPR high-throughput screens.	AI-guided multiplex gene editing (B2M/HLA-E/CD47) overcomes allogeneic rejection (43). CRISPR screens identify *MED12* to enhance CAR-NK potency (45).	Generative AI for *de novo* design of novel immune receptors; “AI design-organoid validation” loops.
Combination Therapy	Fixed combinations based on simple assumptions; ignores cumulative immune toxicities.	Systems biology modeling; ML models predicting multi-cytokine network interactions.	CARMSeD model optimizes multi-antigen CAR designs to overcome antigen escape (61). Synergistic combination of NK-specific checkpoints (e.g., NKG2A) with T cell inhibitors (48).	Integrating Pharmacokinetics/Pharmacodynamics (PK/PD) for “Efficacy vs. Toxicity” dual optimization.
Dosing Regimens	Fixed “3 + 3” dose-escalation and static schedules; overlooks inter-patient heterogeneity.	Multiscale mathematical models combined with Reinforcement Learning (RL).	RL algorithms dynamically adjust combination treatment schedules (62). CURATE.AI platform enables dynamic, personalized dose recommendations (60).	Combining few-shot and transfer learning to explore TME state-based pulsatile dosing paradigms.

Transition from traditional experience-driven methods to AI-driven rational design across three core dimensions of NK cell therapy optimization, including current breakthroughs and future evolutionary pathways.

## 4 From bench to bedside: AI-driven spatial features of NK cells as novel biomarkers

The integration of spatial transcriptomics and AI is giving rise to a next-generation class of clinical biomarkers centered on the spatial ecosystem. Conventional static indicators often focus on individual molecules. In contrast, spatial biomarkers quantify the physical coordinates of NK cells within tissue architecture. They also capture NK cell functional states and interaction networks with neighboring cells. These features may improve clinical predictive performance. This section examines how these spatial biomarkers can transition from the laboratory to the clinic, and the core challenges encountered during this translational process.

### 4.1 Prognostic reconstruction: a paradigm shift from macroscopic staging to microscopic spatial architecture

For decades, tumor prognostic assessment has relied primarily on macroscopic pathological indicators such as TNM staging, which overlooks the complex immune heterogeneity within tumors. The core argument of integrating spatial transcriptomics with AI is that the spatial organizational patterns of immune cells within the TME — rather than mere cell counts — represent the more fundamental determinant of patient clinical outcomes (20). Prognostic prediction in HCC serves as a paradigmatic example of this transition. As detailed in Section 2.1, the TIMES scoring system centered on the spatial distribution of SPON2+ NK cells (**Figure 2B**) has demonstrated prognostic predictive performance in HCC cohorts that significantly surpasses conventional TNM staging (63). Analogous spatial prognostic patterns have been validated across multiple solid tumor types, including NSCLC (19), glioblastoma (20), and colorectal cancer (64), revealing broad applicability. Furthermore, to improve the reproducibility of spatial research, the standardization of spatial metrics is critical. Different studies define “spatial proximity” inconsistently (30 μm vs. 50 μm). To move toward standardization, the field should establish consensus guidelines that define proximity thresholds based on the functional diffusion range of key cytokines (e.g., IL-15, IFN-γ) rather than arbitrary geometric distances, ensuring that spatial metrics reflect true biological interaction potential.

These studies collectively indicate that spatial architecture represents the physical embodiment of cellular functional states. Nevertheless, current research shares three critical limitations. First, most studies adopt retrospective designs and lack prospective validation. Second, the standardization of spatial metrics remains challenging (65). Third, a “knowing–doing gap” persists: although we know that NK cells should localize at the tumor invasive front, how can therapeutic intervention achieve this ideal spatial distribution? This gap constrains the transition of spatial biomarkers from predictive tools to therapeutic guides.

### 4.2 Predicting immunotherapy response: the spatial fingerprint of functional immune cell clusters

The efficacy of immune checkpoint inhibitors (ICIs) depends substantially on whether effector immune cells can form coordinated attacks. Research demonstrates that ICI responders and non-responders exhibit quantifiable and fundamental differences in the spatial organization of the TME (66). Cross-cancer studies consistently indicate that the presence of immune-activating neighborhoods is a key determinant of ICI response. As described in Section 2.1, the immune-activating neighborhood exemplified by the “triad” (NK–cDC1–CD8+ T cells) is significantly associated with ICI response in melanoma, triple-negative breast cancer, and NSCLC (18, 25). In contrast, inhibitory niches are also evident: in HCC, non-responders display greater spatial proximity between regulatory T cells and PD1+ CD8+ T cells (63).

Although existing studies have made substantial progress in revealing microenvironmental heterogeneity, critical limitations remain. First, most studies are constrained by small-scale, single-center designs — whether the predictive performance of the “triad” spatial structure remains robust in large-scale, multicenter cohorts is yet to be established. Second, the inconsistency of spatial interaction scoring standards (67) impedes standardized clinical implementation. At a deeper level, current research remains at the correlational stage: are immune-activating neighborhoods the cause or the consequence of ICI response? Answering these questions requires the deep integration of spatial biomarker research with therapeutic strategy development, advancing from “passive prediction” to “active intervention”.

### 4.3 Virtual spatial omics: AI-driven democratization of computational pathology

Although high-throughput spatial technologies provide high-dimensional spatial information, their high cost and technical complexity represent the primary barriers to clinical adoption. virtual spatial omics stands as one of the most translationally promising breakthroughs to date. As shown in **Figure 2C**, the HEX model published by Li et al. in Nature Medicine (2026) accurately predicts biomarker expression from routine H&E images, achieving an AUC of 0.85 for immunotherapy response prediction — significantly outperforming conventional PD-L1 detection (28). The potential clinical value of virtual spatial omics lies in its scalability. Hospitals equipped with pathology scanners may be able to extract molecularly relevant information from existing H&E slides through AI. This approach does not require additional costly molecular assays. Therefore, it may lower the access threshold for precision medicine at a relatively low marginal cost.

Nevertheless, virtual spatial omics confronts critical challenges. First, the black-box nature of these models renders their predictions difficult to interpret (68), potentially limiting clinician trust. Second, predictive accuracy is highly dependent on the quality and diversity of training data — if training data predominantly originate from European and American populations, model performance in Asian populations may be substantially compromised. Finally, the generalizability of these models to untrained cancer types or rare histological subtypes requires validation.

### 4.4 Challenges on the translational path

Advancing AI-driven spatial biomarkers from research discovery to routine clinical application still requires overcoming barriers spanning technical, economic, and regulatory dimensions. Current spatial omics platforms differ substantially in data type, resolution, and sensitivity. As a result, AI models developed on one platform may show performance degradation when applied to another platform. This problem is largely related to domain shift (69). Furthermore, there is no unified gold standard for the full workflow of spatial omics. This workflow includes sample processing, data generation, and data analysis. The lack of standardization poses a substantial challenge to the reproducibility of multicenter studies (67, 70). Plummer et al. (2025) emphasized that spatial transcriptomics lacks standardized metrics for evaluating technical performance across sites (65). High economic costs also constitute a major obstacle: the cost of a single spatial transcriptomics slide can reach thousands of dollars, far exceeding that of conventional immunohistochemistry (approximately $50–200 per sample). When combined with the requisite sequencing instruments, computational servers, and specialized personnel, spatial omics is unlikely to become a universally accessible diagnostic tool in public healthcare systems in the near term.

A stringent regulatory approval pathway presents another major challenge. In the United States, AI/ML-driven diagnostic software is typically classified by the FDA as Software as a Medical Device (SaMD), and complex models intended to assist treatment decisions are frequently placed under the highest-risk regulatory tier. This means that any spatial biomarker seeking market approval must rigorously demonstrate clinical validity through large-scale, multicenter, prospective clinical trials. Zhang et al. (2025) noted that the FDA approved over 1,000 AI medical devices between 2015 and 2025, yet 96% were approved through the 510(k) pathway, which has limited capacity to accommodate the approval of innovative biomarkers (71). Aggarwal et al. further emphasized that current AI diagnostic tools face a significant regulatory approval bottleneck (68). Only by successfully overcoming these three barriers — technical, economic, and regulatory — can precision diagnostics based on NK cell spatial niches move toward clinical translation after validation. This process requires coordinated efforts across multiple stakeholders. Technology developers should prioritize cross-platform compatibility and interpretability. Regulatory agencies need to establish appropriate approval pathways for AI-assisted spatial biomarkers. Healthcare insurance systems should consider reasonable reimbursement policies. The academic community also needs to promote standardized metrics. More importantly, the value of spatial biomarkers lies not only in more accurate prediction, but also in actionable therapeutic guidance. Future research should deeply integrate spatial biomarker development with therapeutic strategy design, achieving a closed-loop from diagnosis to intervention.

Although deep learning models demonstrate potential that surpasses human experts in decoding NK cell spatial niches, the “black-box” nature of their internal operations remains the core barrier impeding broad clinical adoption. To address this challenge, explainable artificial intelligence (XAI) is translating complex nonlinear algorithms into intuitive biological interpretations through techniques such as Local Interpretable Model-agnostic Explanations (LIME) and SHapley Additive exPlanations (SHAP). In a 2025 study on NSCLC immunotherapy response, researchers used SHAP to identify and quantify the contribution of spatial neighborhood features within the TME to prediction outcomes. This analysis allowed clinicians to examine how the model generated a “high response” judgment. In particular, the judgment was related to the physical proximity of NK cells and cDC1 cells (72). Furthermore, the FDA released updated guidelines for AI-driven medical devices in 2026, emphasizing the role of model transparency in safety assessment. In this context, systems with real-time interpretability capabilities may be particularly relevant. Examples include pathology whole-slide image (WSI) analysis platforms integrated with explainable modules. Such systems could support multidisciplinary tumor teams (MDTs) when evaluating NK cell adoptive therapy dosing regimens (73). This shift from “delivering results” to “explaining logic” not only strengthens clinician trust by mitigating algorithmic bias, but also provides the ethical and technical foundation for the transition of NK cell therapy from experimental research to standardized clinical practice.

## 5 Conclusions and future perspectives

The integration of spatial transcriptomics and AI is contributing to a shift in NK cell immunotherapy research by enabling more detailed characterization of TME. Rather than viewing the TME only as a collection of discrete cellular components, spatially resolved approaches allow researchers to examine immune cell localization, neighborhood relationships, and structural organization within tissue contexts (18, 74). A recurring theme of this review is the potential transition from response prediction toward treatment optimization, although this transition remains dependent on further mechanistic, technical, and clinical validation.

At the biomarker level, the TIMES scoring system derived from spatial transcriptomics and virtual spatial omics approaches based on computational pathology, such as the HEX model, have shown predictive value across retrospective cohorts (28, 63). These findings suggest that the anti-tumor activity of NK cells may be influenced not only by their absolute abundance, but also by the specific spatial microenvironment in which they are located (64, 66). Nevertheless, an important limitation of current research is that many findings remain correlational rather than causal. This gap between identifying spatial associations and using them to guide therapeutic intervention limits the current clinical utility of spatial biomarkers. AI-assisted therapeutic strategies may help address this gap by supporting CAR-NK cell engineering, combination therapy selection, and dosing optimization. However, these applications should be viewed as emerging decision-support approaches rather than established clinical solutions, and their value will require prospective validation in well-designed clinical studies.

**Figure 4:**
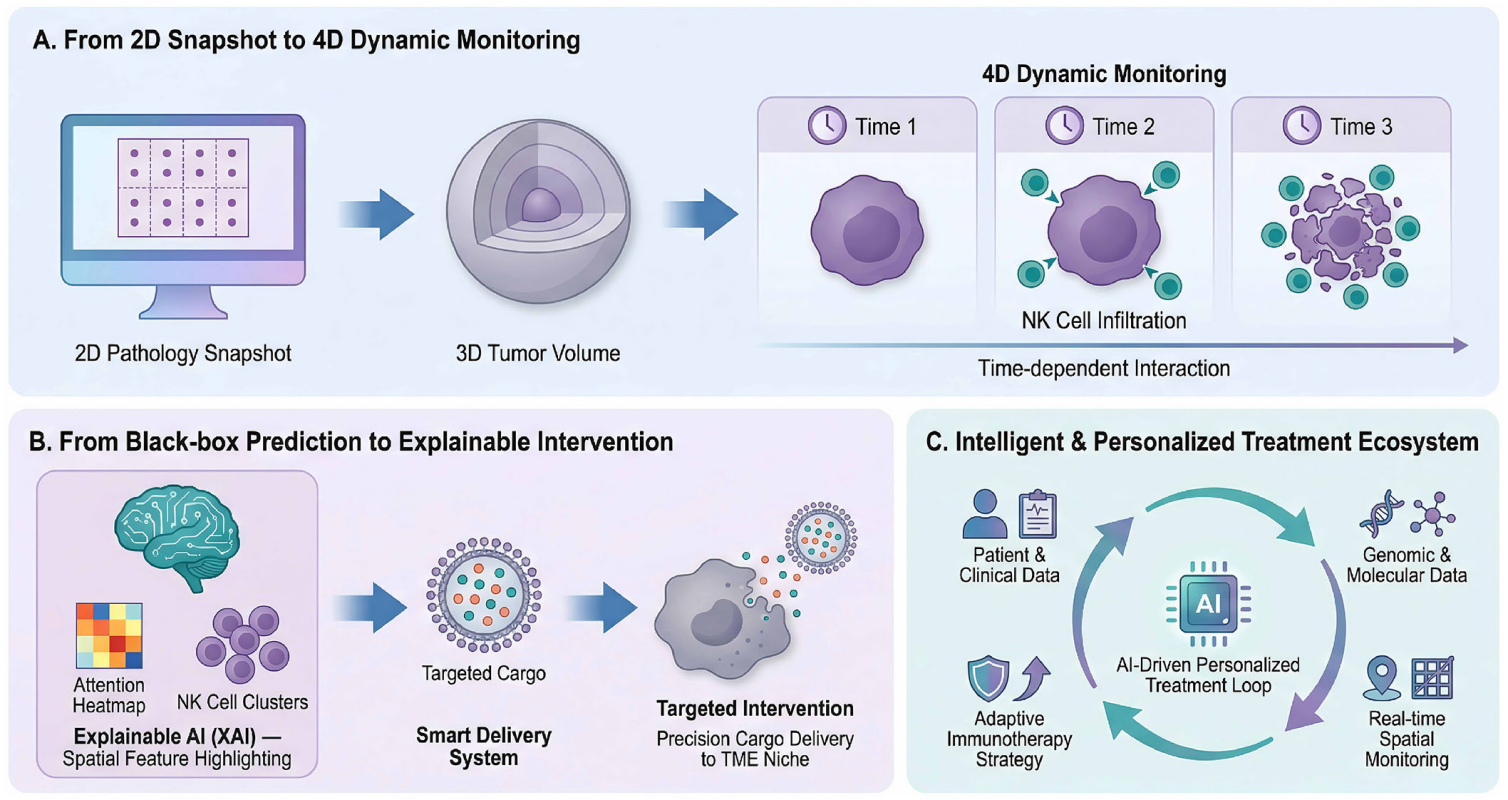
Future perspectives of NK cell immunotherapy. **(A)** Evolution from 2D spatial snapshots to 4D dynamic monitoring of TME remodeling. **(B)** Explainable AI (XAI) models, such as SHAP, elucidating underlying spatial features to guide targeted delivery hubs. **(C)** The ultimate closed-loop ecosystem of personalized and adaptive NK cell immunotherapy.

Looking ahead, three areas may be particularly important for the further development of AI-assisted NK cell immunotherapy. First, spatial analysis may move from two-dimensional tissue sections toward three-dimensional and longitudinal models, as illustrated by the trajectory in **Figure 4A**. At present, most spatial omics studies remain based on thin tissue sections of 5–20 μm (75), which provide only limited cross-sectional information from three-dimensional tumors and may introduce sampling bias (76, 77). Emerging technologies for thick tissue analysis, such as Deep-STARmap for tissue blocks up to 200 μm thick (78) and Open-ST for high-resolution three-dimensional profiling (79), may help reconstruct the spatial organization of the TME more comprehensively. When combined with longitudinal biopsy sampling, these approaches could support the development of four-dimensional models of TME evolution under therapeutic pressure (80, 81), although their scalability, reproducibility, and clinical feasibility remain to be established.

Second, the interpretability of AI models will be essential for translating spatial biomarkers into clinical contexts. Current AI models often face the “black-box” problem, in which predictive performance may be observed but the biological basis of model decisions remains difficult to interpret (68). Future studies should further integrate explainable AI techniques, including attention mechanisms, feature importance analysis, and causal inference, into spatial omics analyses (82, 83) (**Figure 4B**). By identifying spatial features that are most strongly associated with model predictions, these approaches may help generate testable hypotheses for therapeutic intervention, such as targeting cancer-associated fibroblast barriers or developing delivery systems for immune agonists, including NK cell engagers, within specific immune activation regions (54, 84). However, whether such strategies can reliably remodel the TME and improve clinical outcomes remains to be demonstrated.

Third, the clinical translation of spatial biomarkers will require coordinated progress across technical, economic, and regulatory domains. Spatial omics platforms currently differ in resolution, sensitivity, data structure, and analytical workflows, which may limit cross-platform reproducibility and model generalizability. Technology developers will need to improve domain adaptation and transfer learning methods to reduce systematic differences across platforms (70, 85). At the same time, regulatory agencies, healthcare systems, and the academic community will need to establish appropriate validation frameworks, reimbursement strategies, standardized metrics, and multicenter prospective studies (65). Without these supporting conditions, spatial biomarkers are unlikely to become routine clinical tools, even if their predictive performance is promising in research settings.

Overall, AI-assisted analysis of spatial and multi-dimensional patient data may contribute to more precise characterization of NK cell states, therapeutic response patterns, and treatment-relevant features within the TME. In the future, such approaches could support a more adaptive framework for NK cell immunotherapy, involving biomarker-guided patient stratification, CAR-NK cell design, combination therapy selection, dosing adjustment, and longitudinal response monitoring, as conceptually summarized in **Figure 4C**. However, this framework should be considered an emerging research direction rather than an established clinical model. Its successful implementation will depend on prospective validation, biological interpretability, cross-center reproducibility, regulatory acceptance, and demonstration of clinically meaningful benefit.
